# *Renius cornutus*, a new genus and species of Chilocorini from Tibet, China (Coleoptera, Coccinellidae)

**DOI:** 10.3897/zookeys.678.11862

**Published:** 2017-06-07

**Authors:** Wenjing Li, Lizhi Huo, Dirk Ahrens, Shunxiang Ren, Xingmin Wang

**Affiliations:** 1 Key Laboratory of Bio-Pesticide Innovation and Application, Engineering Technology Research Center of Agricultural Pest Biocontrol, Guangdong Province; Department of Entomology, South China Agricultural University, Guangzhou 510640, China; 2 Zoologisches Forschungsmuseum Alexander Koenig Bonn, Adenauerallee 160, 53113 Bonn, Germany

**Keywords:** China, Coccinelloidea, Coleoptera, new genus, new species, Tibet

## Abstract

A new monotypic genus of Chilocorini, *Renius* Li & Wang, **gen. n.**, with a new species *R. cornutus* Li et Wang, **sp. n**. is described from Tibet, China. All diagnostic features are illustrated. The relationships with other genera of Chilocorini are discussed and a key to Chinese genera of Chilocorini is provided.

## Introduction

Ladybirds belonging to tribe Chilocorini, well-known primary predators of coccids, with known instances of aphidophagy (Giorgi et al. 2009), are distributed worldwide. Chilocorini consists of 26 genera and 280 species (Łączyński and Tomaszewska 2012), classified under the subfamily Chilocorinae with Platynaspidini and Telsimini (Sasaji 1968). However, recent molecular phylogenetic analyses reveal that Chilocorinae does not represent a monophyletic group (Giorgi et al. 2009; Margo et al. 2010; Seago et al. 2011; Robertson et al. 2015), and Chilocorini should be classified in the subfamily Coccinellinae (Ślipiński 2007 and Seago et al. 2011).

The tribe is also diverse in China being represented with nine genera and 39 species (Pang et al. 2004; Ren et al. 2009; Hu et al. 2013; Li et al. 2015a; 2015b). During our study of the Chinese Chilocorini, a particular species from Tibet was found, recorded twice during collecting trips in 2009 and 2011, respectively. This new species is similar to members of *Orcus* Mulsant, 1850 in body shape and coloration. However, some characters make it hard to group it to any extant genus of Chilocorini.

Therefore, a new genus, *Renius* gen. n. is established for the only so far known species, *Renius cornutus* Li et Wang sp. n., which is also described here. Additionally, a key is provided to the genera of Chilocorini known in China and the diagnostic features of the new genus and species are illustrated. Lastly, the relationships between *Renius* and the other genera of Chilocorini are discussed.

## Materials and methods

Specimens examined in this study were collected in Tibet, China. Type specimens designated in the present paper are deposited at the Department of Entomology, South China Agriculture University (**SCAU**), Guangzhou.

All the morphological photographs were taken by Zeiss AxioCam HRc digital camera mounted on a Zeiss Stereo Discovery V20 stereomicroscope or Zeiss Imager Z2m microscope. A number of serial images were combined in Zerene Stacker in order to obtain an entirely focused image, and photographs were cleaned up and laid out in plates in Adobe Photoshop CS 8.0. Morphological terms of Coccinellidae follow Ślipiński (2007) and Ślipiński and Tomaszewska (2010). The following measurements were made with an ocular micrometer:

**TL** total length, length from apical margin of clypeus to apex of elytra;

**TW** total width, width across both elytra at widest point;

**TH** height measured across the highest point of the elytra;

**HW** head width in frontal view, including eyes;

**PL** pronotal length, from middle of anterior margin to base of pronotum;

**PW** pronotal width at widest point;

**EL** elytral length, from the apex of the elytra to the base including the scutellum;

**EW** elytral width, equal TW.

## Results

### Renius

Taxon classificationAnimaliaColeopteraCoccinellidae

Li & Wang gen. n.

http://zoobank.org/FA483030-8C57-40AC-B403-06301177A11A

#### Type species.

*Renius cornutus* Li & Wang, sp. n.

#### Diagnosis.

**Figure 1.**
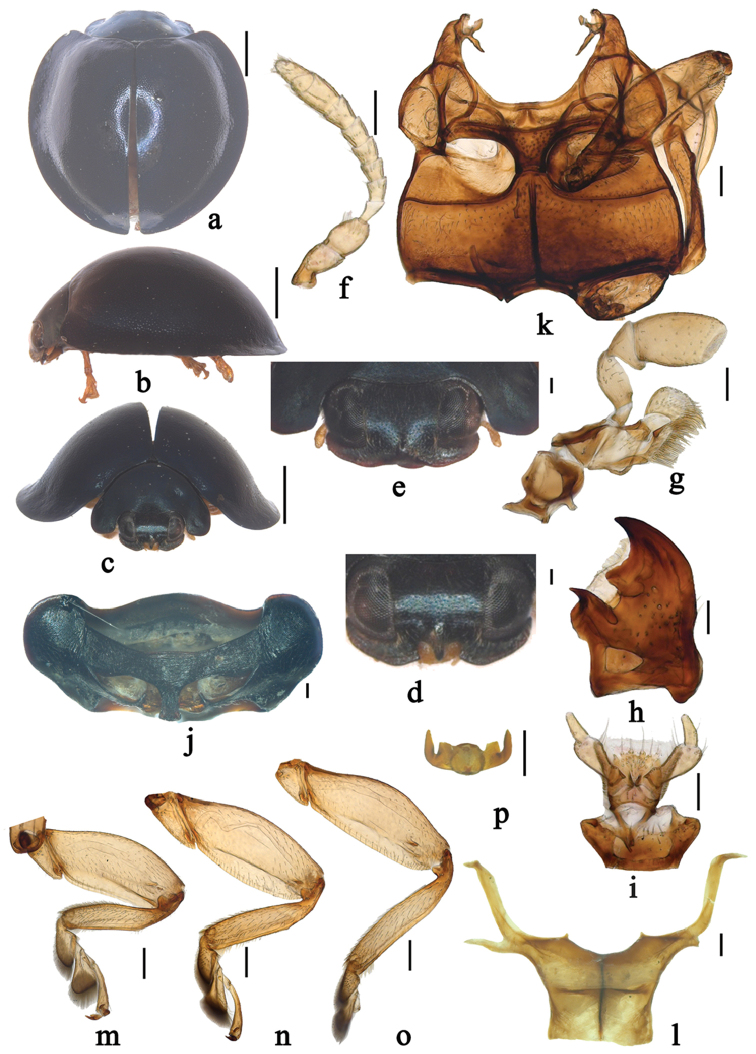
*Renius cornutus* Li et Wang, sp. n. **a** dorsal view **b** lateral view **c–d** frontal view, male **e** frontal view, female **f** antenna **g** maxilla **h** mandible **i** labium **j** prothorax, ventral view **k** meso- and metaventrite **l** metendosternite **m** front leg **n** mid leg **o** hind leg **p** tarsal claws. Scale bars 1.0mm **a–c**, 0.1 mm **d–p**.

**Figure 2.**
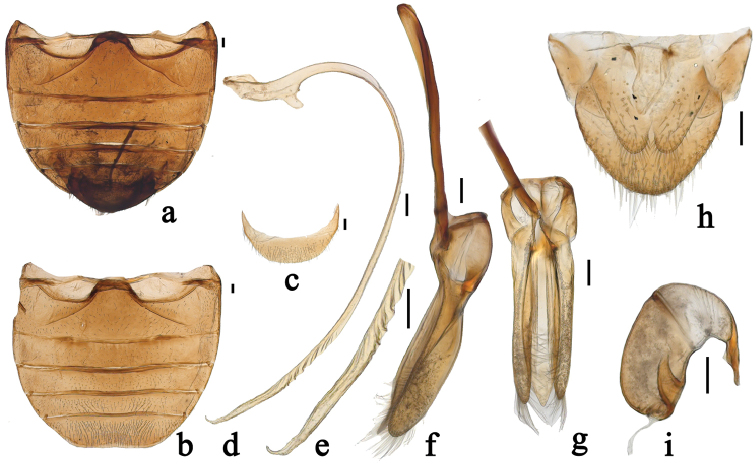
*Renius cornutus* Li et Wang, sp. n. **a** abdomen (male) **b** abdomen (female) **c** abdominal ventrite 6, female **d** penis **e** apex of penis **f** tegmen, lateral view **g** tegmen, ventral view **h–i** female genitalia: **h** ovipositor **i** spermatheca. Scale bars 0.1 mm.

The genus *Renius* can be distinguished from other genera of the tribe Chilocorini by the following combination of characters: clypeus distinctly projecting medially in male (Fig. 1c–d), slightly projecting medially in female (Fig. 1e); antenna composed of 10 antennomeres (Fig. 1f); basal margin of pronotum with distinct border line; prosternal process long, narrow, parallel sided, without carina (Fig. 1j); outer elytral margin strongly reflexed without distinct bead; epipleuron without foveae for the reception of mid and hind legs; abdominal postcoxal lines complete or almost complete, arcuate (Fig. 2a–b); legs with stout femora, tibiae slender without apical spurs (Fig. 1m–o); between the coxites with large, subtriangular sclerite (Fig. 2h).

#### Description.

Body broadly rounded, moderately convex, dorsum and underside glabrous (Fig. 1a). Head large, 0.54–0.56 times pronotal width, covered with short, yellow pubescence. Eyes large, approximately oval, densely faceted, with inner sides subparallel. Clypeus with distinct horn-like projection, both sides of projection with a small subquadrate gap in male (Fig. 1c–d), in female clypeus with weak projection medially, without any gap (Fig. 1e). Antenna 10-segmented, scape asymmetrical, narrow at basal 1/3, distinctly expand to apical 2/3, pedicel subquadrate, with the same width as scape anteriorly, antennomeres 3–5 gradually broadening and shortening, 5–8 gradually broadening with the same length, antennomere 9 distinctly longer and wider than antennomere 8, terminal antennomere slightly narrower and shorter than antennomere 9, truncate and oblique at apex (Fig. 1f). Mandible unidentate, prostheca distinct, outer margin of mandible slightly curved (Fig. 1h). Terminal maxillary palpomere elongate with sides slightly expanded, apex obliquely truncate (Fig. 1g). Penultimate labial palpomere stout, 1.5 times as wide as and longer than terminal labial palpomere; terminal labial palpomere subconical (Fig. 1i).

*Prothorax* descending anteriorly (Fig. 1b–c). Basal margin of pronotum with visible border line. Prosternum T-shaped, in front of coxae distinctly longer than basal width of prosternal process; prosternal process long, narrow, parallel sided, without carina (Fig. 1j). Mesoventrite approximately trapezoidal, with anterior margin straight. Meso and metaventral process narrow, junction straight, with visible suture (Fig. 1k). Postcoxal lines on metaventrite descending laterally. Tendons of metendosternite separated by much less than width of stalk and placed close to middle (Fig. 1l). Scutellum small and triangular. Elytra distinctly wider than pronotum at base; outer elytral margin strongly reflexed without distinct bead; elytral epipleuron distinctly broaden with descending outer portion, without grooves. Abdomen with six ventrites in both sexes; abdominal postcoxal lines complete or almost complete, arcuate (Fig. 2a–b). Legs with stout femora, tibiae slender, without tibial spurs (Fig. 1m–o); tarsal claws stout, with trapezoidal basal tooth, about 1/2 length of claw (Fig. 1p).

#### Etymology.

The generic name is dedicated to the memory of the well-known Chinese entomologist, Ren Shunxiang, who devoted most of his life to the study of Coccinellidae and biological control. Gender masculine.

### Renius cornutus

Taxon classificationAnimaliaColeopteraCoccinellidae

Li & Wang sp. n.

http://zoobank.org/BE549488-ED63-44FE-9BB6-26BBD7782787

Figs 1 , 2

#### Type material.

Holotype. male, CHINA: Tibet: Gedang, Motuo County, [29°27.49'N; 95°32.57'E], *ca.* 1600m, 11.v.2011, Huo LZ leg (SCAU). Paratypes. The same information as the holotype; 1 female, Tibet: Beibeng to Hanmi, Motuo County, [29°19.23'N; 95°07.21'E], 800–2100m, 5–8.v.2011, Huo LZ leg (SCAU); 1 female, Tibet: No. 1 Bridge, Hanmi, Motuo County, [29°21.06'N; 95°04.53'E], *ca.* 2000m, 14.v.2009, Wang XM leg (SCAU).

#### Diagnosis.

*Renius cornutus* can be easily distinguished from other species of the tribe Chilocorini by the roundish body with bluish black pronotum and elytra; medially projecting clypeus and subtriangular sclerite between coxites.

#### Description.

TL: 4.43–5.00 mm, TW: 4.56–5.00 mm, TH: 1.94–2.31 mm, TL/TW: 0.97–1.00, PL/PW: 0.46–0.47, EL/EW: 0.77–0.81.

*Body* roundish, moderately convex. Head bluish black, mouthparts yellow, only clypeal portion covered with short, greyish pubescence. Pronotum, scutellum and elytra bluish black (Fig. 1a–d). Underside bluish black except legs yellow, abdomen brownish black, sparsely covered with short, grayish pubescence.

*Head* relatively large, 0.54–0.56 times pronotal width, punctures on frons large and moderately densely distributed, 0.5–2.0 diameters apart, surface polished between punctures. Eyes approximately oval, densely faceted, widest interocular distance about 0.46 times head width (Fig. 1c–d). Pronotum 0.49–0.50 times elytral width, pronotal punctures large and unevenly distributed, similar to those on head, 1.0–3.5 diameters apart, lateral punctures sparser than those on disc, surface polished between punctures. Punctures on elytra large and densely distributed, 1.0–2.0 diameters apart, similar to those on pronotum. Elytral epipleura very wide, descending without groove. Abdominal postcoxal lines complete or almost complete, arcuate; posterior margin of male abdominal ventrite 5 weakly emarginate and ventrite 6 rounded; posterior margin of female abdominal ventrite 5 straight and ventrite 6 rounded (Fig. 2a–c).

*Male genitalia*: penis slender, penis capsule with long outer arm and short inner one, apex of penis acute, screw-shaped from apical 1/11 to apical 2/11 (Fig. 2d–e). Tegmen stout, penis guide narrow at base, widest at basal 1/3 then gradually converging to blunt tip, symmetrical in ventral view; widest at base gradually converging to blunt apex in lateral view. Parameres stout, slightly shorter than penis guide, densely covered with short setae at inner surfaces and a distal end in lateral view (Fig. 2f–g).

*Female genitalia*: ovipositor with coxites elongate, approximately triangular, with large, subtriangular sclerite between coxites, 2/3 time as long as coxites (Fig. 2h); styli absent. Spermatheca oblong-oval, stout, with long and slender appendage at apex (Fig. 2i).

#### Distribution.

Motuo County, Tibet, China.

#### Etymology.

The species name is derived from Latin *cornutus* and refers to middle of clypeus with a horn-like projection in the male.

### Key to the Chinese genera of Chilocorini

**Table:** 

1	Meso- and metatibia with apical spurs	**2**
–	Meso- and metatibia without apical spurs	**8**
2	Antenna composed of less than 10 antennomeres	**3**
–	Antenna composed of 10 antennomeres	**4**
3	Antenna with 8 antennomeres	***Brumoides* Chapin**
–	Antenna with 9 antennomeres	***Chujochilus* Sasa ji**
4	Pronotal basal margin with bordering line	**5**
–	Pronotal basal margin without bordering line	***Xanthocorus* Miyat ake**
5	Base of pronotum and elytra not contiguous all along their length	**6**
–	Base of pronotum and elytra contiguous all along their length	***Priscibrumus* Kovář**
6	Tarsal claw with distinct subquadrate or triangulate basal tooth	**7**
–	Tarsal claw simple, sometimes thickened at base	***Brumus* Mulsant**
7	Prosternal process rounded at apex; abdominal postcoxal lines narrowly open laterally	***Parexochomus* Barovsky**
–	Prosternal process truncate at apex; abdominal postcoxal lines closed laterally	***Exochomus* Redtenbacher**
8	Antenna with 8 antennomeres	**9**
–	Antenna with 10 antennomeres	***Renius* Li et Wang, gen. n.**
9	Terminal maxillary palpomere slender and elongate, approximately 3 times as long as basal width	***Phaenochilus* Weise**
–	Terminal maxillary palpomere stout, from 1 to 2 times as long as basal width	***Chilocorus* Leach**

## Discussion

Although *Renius* share ten antennomeres of the antenna and similar shapes of the tibiae with *Exochomus*, it does not have the terminal antennomere embedded in the penultimate one, and it lacks apical spurs on mid and hind tibiae.

Initially, *the specimens* were thought as a member of *Orcus* Mulsant. Both genera share many characters, like: roundish body; basal margin of pronotum with border line; elytral margin strongly reflexed; elytral epipleuron broadened; penultimate labial palpomere stout; shape of abdominal postcoxal lines etc. (Łączyński and Tomaszewska 2009). Therefore, it appears likely having a close relationship with *Orcus* Mulsant. However, the shape of spermatheca, mesoventrite, and meso-metaventral junction of *Renius* resemble those of *Chilocorus* Leach. According to morphology, *Renius* should have a closer relationship with *Orcus* rather than with *Chilocorus*. However, the molecular phylogenetic analysis of Chilocorini reveal *Renius* and (*Chilocorus* + *Phaenochilus* + *Anisorcus*) to be a sister group (Li et al., in prep.).

*Renius* differs from all other genera of Chilocorini in having unique characters, such as clypeus with median projection and subtriangular large sclerite between coxites of female ovipositor. These characters, together with an antenna composed of ten antennomeres with antennomeres 3–5 gradually shortening, define this new genus.

## Supplementary Material

XML Treatment for Renius

XML Treatment for Renius cornutus

## References

[B1] Hu SC Lin XW Wang BH (2013) Coccinellidae of The Qinghai-Xizang Plateau. Henan Science and Technology Press, Zhengzhou, 213 pp.

[B2] Giorgi JA Vandenberg NJ Mchugh JV Forrester JA Ślipiński SA Miller KB Shapiro LR Whiting MF (2009) The evolution of food preferences in Coccinellidae. Biological Control 51: 215–231. https://doi.org/10.1016/j.biocontrol.2009.05.019

[B3] Łączyński P Tomaszewska W (2009) A review of the genus *Orcus* Mulsant (Coleoptera: Coccinellidae: Chilocorini). Annales Zoologici 59: 585–611. https://doi.org/10.3161/000345409X484955

[B4] Łączyński P Tomaszewska W (2012) *Chapinaria*, new genus of Chilocorini for *Endochilus meridionalis* Sicard from Africa (Coleoptera: Coccinellidae). Annales Zoologici 62: 1–9. https://doi.org/10.3161/000345412X633658

[B5] Li WJ Chen XS Wang XM Ren SX (2015a) Contribution to the genus *Xanthocorus* Miyatake (Coleoptera, Coccinellidae, Chilocorini). ZooKeys 511: 89–98. https://doi.org/10.3897/zookeys.511.9584 10.3897/zookeys.511.9584 PMC4523747 26257553

[B6] Li WJ Huo LZ Wang XM Chen XS and Ren SX (2015b) The genera *Exochomus* Redtenbacher, 1843 and *Parexochomus* Barovsky, 1922 (Coleoptera: Coccinellidae: Chilocorini) from China, with descriptions of two new species. The Pan-Pacific Entomologist 91(4): 291–304. https://doi.org/10.3956/2015-91.4.291

[B7] Magro A Lecompte E Magne F Hemptinne J Crouau-Roy B (2010) Phylogeny of ladybirds (Coleoptera: Coccinellidae): are the subfamilies monophyletic? Molecular Phylogenetics and Evolution 54: 833–848. https://doi.org/10.1016/j.ympev.2009.10.022 19903531 10.1016/j.ympev.2009.10.022

[B8] Mulsant ME (1850) Species des Coléoptères Trimères Sécuripalpes. Annales des Sciences Physiques et Naturelles, d’Agriculture et d’Industrie, publiées par la Société nationale d’Agriculture, etc. , de Lyon, Deuxième Série, 2, 1104 pp. [part 1, 1–450; part 2, 451–1104]

[B9] Pang H Ren SX Zeng T Pang XF (2004) Biodiversity and their utilization of Coccinellidae in China. Science and Technology Press of Guangdong, Guangzhou, 168 pp. [In Chinese]

[B10] Robertson JA Ślipiński A Moulton M Shockley FW Giorgi A Lord N Mckenna DD Tomaszewska W Forrester J Miller KB Whiting MF Mchugh J (2015) Phylogeny and classification of Cucujoidea and the recognition of a new superfamily Coccinelloidea (Coleoptera: Cucujiformia). Systematic Entomology 40: 745–778. https://doi.org/10.1111/syen.12138

[B11] Ren SX Wang XM Pang H Peng ZQ Zeng T (2009) Colored pictorial handbook of ladybird beetles in China. Science Press, Beijing, 336 pp. [In Chinese]

[B12] Sasaji H (1968) A revision of the Formosan Coccinellidae (II) tribes Stethorini, Aspidimerini and Chilocorini (Coleoptera). Etizenia, Fukui 32: 1–24.

[B13] Seago AE Giorgi JA Li JH Ślipiński A (2011) Phylogeny, classification and evolution of ladybird beetles (Coleoptera: Coccinellidae) based on simultaneous analysis of molecular and morphological data. Molecular Phylogenetics and Evolution 60: 137–151. https://doi.org/10.1016/j.ympev.2011.03.015 21426943 10.1016/j.ympev.2011.03.015

[B14] Ślipiński A (2007) Australian ladybird beetles (Coleoptera: Coccinellidae), their biology and classification. ABRS, Canberra, 286 pp.

[B15] Ślipiński A Tomaszewska W (2010) Coccinellidae Latreille, 1802. In: Leschen RAB Beutel RG Lawrence JF (Eds) Handbook of Zoology, Vol. 2, Coleoptera. Walter de Gruyter GmbH & Co. KG, Berlin/New York, 454–472.

